# Austerity Talk and Crisis Narratives: Memory Politics, Xenophobia, and Citizenship in the European Union

**DOI:** 10.3389/fsoc.2020.00014

**Published:** 2020-03-13

**Authors:** Helga K. Hallgrimsdottir, Ari Finnsson, Emmanuel Brunet-Jailly

**Affiliations:** ^1^School of Public Administration, University of Victoria, Victoria, BC, Canada; ^2^Social and Political Thought, York University, Toronto, ON, Canada

**Keywords:** Europe, economic crises, memorialisation, citizenship, austerity and culture

## Abstract

The focus in this paper is on understanding the complex intersections between crises and memory politics in shaping conversations about citizenship through an examination of the two defining crises of our time: the global financial crisis (GFC) of 2008 and the migrant crisis in the European Union (starting in 2011 and continuing). The paper looks at these crises as narrative devices that intersect with memory politics in ways that heighten and intensify xenophobic and nationalist anxieties. The paper's discussion is primarily theoretical, complemented with evidence drawn from public statements and policy platforms of three key right-wing Eurosceptic parties in the United Kingdom, France, and Germany (the United Kingdom Independence Party (UKIP), the Rassemblement National (RN), and the Alternativ für Deutschland (AfD).

## Introduction

Crisis is the catchword of our current historical moment (Dinan et al., 2017). For residents of the European Union, the global economic and financial crisis (GFC) of 2008 was succeeded by the sovereign debt crisis in 2009 (the Eurozone crisis), and the refugee reception (or migrant)^1^ crisis in 2015. The most recent European crisis, as evidenced by the electoral success of far-right Eurosceptic parties in the latest European elections in 2019, and the Brexit referendum in 2016 (Lapavitsas et al., 2010; Bulmer, 2014), concerns the very question of an integrated European Union.

While it is important to recognize differences between the crises listed above, they do share at least one significant commonality: they all invoke foundational challenges to the nature of belonging, inclusion and integration in the European Union—that is, to citizenship (see for instance Virdee and McGeever, 2018). There are many manifestations of this: for one, the Eurozone crisis led to a growing cleavage between core and periphery EU nations that shattered faith in the ability of EU institutions to mitigate inequalities between nations (Magone, 2015). As argued by Murray and Longo, while the debate about the democratic deficit in the EU has long historical roots, this debate is now conflated with charges of solidarity and social justice deficits in the EU (Murray and Longo, 2015) reflecting again a fundamental challenge to the notion and promise of EU citizenship as an integrative status and guarantor of social rights.

At the same time, European citizenship is itself a fraught concept, and historically laden with diverse (and often conflicting) memorializations of Europe's past (Judt, 2006). In other words, whereas recent European political, economic, and social crises have triggered conversations about^1^ European belonging, these conversations necessarily take place against the backdrop of European memory politics; as articulated by Tony Judt, Europe is “forever mortgaged to its past” (Judt, 2006, p. 831). Layered onto memory politics, citizenship both hinders and enables European integration.

The focus in this paper is on understanding the complex intersections between crises and memory politics in shaping conversations about citizenship through an examination of the two defining crises of our time: the global financial crisis (GFC) of 2008 and the refugee reception crisis in the European Union (starting in 2011 and continuing). The paper looks at these crises as narrative devices that intersect with memory politics in ways that heighten and intensify xenophobic and nationalist anxieties. The discussion presented in this paper is primarily theoretical, complemented with evidence drawn from public statements and policy platforms of three key right-wing Eurosceptic parties in the United Kingdom, France, and Germany (the United Kingdom Independence Party (UKIP), the Rassemblement National (RN), and the Alternativ für Deutschland (AfD). These parties were chosen because of their explicit engagement with European integration politics and narratives in their political activities; the focus below is on how these parties narrated the GFC and the refugee reception crisis, and how these narratives drew on and generated certain understandings of European Union citizenship.

The paper begins with a brief examination of the GFC and the refugee reception crises as narrative challenges to citizenship, followed by a discussion on the intersections between citizenship and memory politics. A key goal of this section is to pull out not only how citizenship functions as a construct for understanding the complex relationships between states and individuals, but also how citizenship can simultaneously call forth exclusionary and inclusionary narratives of social, economic, and political belonging. This is followed by a discussion of “crisis” as an empirical, normative, and narrative construct; and as one of the defining narrative constructs of our current era. This is followed by a brief empirical illustration, using examples of how framings of the economic crisis and the refugee reception crisis by right-wing Eurosceptic parties, intersect with reigning memory politics. The paper concludes by suggesting that one of the most pressing challenges the EU today faces concerns resisting the urge to manage crises (whether political, economic, or demographic) by thinning citizenship (Stasiulis, 2004).

## The 2008 Economic Meltdown and the Refugee Reception Crisis in the European Union

While the first two decades of this century have been dominated by various crisis narratives (including in particular climate crises and political crises), the focus here is on two crises that have most immediately preceded the current challenges facing European Union integration: the global financial crisis (GFC) and the refugee reception crisis.

The GFC is generally dated to the fall of 2008 when the worst financial crisis since the Great Depression swept across the globe (Crotty, 2009). Failures in the US subprime mortgage market in late 2007 triggered a series of other bank failures, leading to a crisis that then spread through the global financial market, giving rise to deep recessions across much of the OECD countries. Much has been written on the economics of the 2008 global financial crisis, as well as of the global economic downturn that followed in its wake (Crotty, 2009; Rose and Spiegel, 2011; Stockhammer, 2015; Stiglitz, 2016). Recovery from the GFC was slow and varied considerably across the EU (Antoshin et al., 2017); in addition, the GFC solidified the dominance of austerity policies and discourse as the only valid response to economic downfall, and, in many of the worst affected countries, led to significant cutbacks to social services, a general retrenchment of the welfare state and wage stagnation (Karger, 2014; Mitchell and Flanagan, 2014).

More than a decade later, the disruptive effects of the economic crisis are still playing out across the EU. On the one hand, there has been a resurgence of nationalist politics, including both ultra-right wing parties as well as the mainstreaming of populist nationalist ideas within established parties (Melzer and Serafin, 2013; Vasilopoulou and Halikiopoulou, 2015). At the same time, activists from both the right and left have parlayed the financial crisis and the policy responses to it into a narrative that renders mainstream macro-economics, as well as liberal democracy itself, as morally and intellectually bankrupt (Fukuyama, 2012; Serricchio et al., 2013). The Brexit referendum results, the rhetoric used by the far-right and far-left candidates in, for instance the French, Dutch, and German elections of 2017, and the Belgian, Italian and Greek, and Hungarian elections of 2018, alongside of the waning influence of social-democratic ideals in nations on the periphery of the EU, such as Turkey, point more specifically to an emerging normative crisis with regards to the European integration project that in the face of increased security demands undermines European Union solidarity (Bruszt, 2015). The rise of populist politics, on both the left and the right, appears to be the most significant legacy of the GFC. A large-scale comparative study commissioned by the *Guardian*, showed recently that the proportion of Europeans voting for populist parties has risen from about 15% in 2008 to over 25% in 2018 (Lewis et al., 2018). This trend has to be viewed within the context of austerity policies, welfare state retrenchment, and rising inequality in Europe. It is also important to identify the formation of nationalist parties as not just an outcome but also a cause of rising nationalist sentiments.

This recent rise of nationalist and xenophobic politics in the EU is embedded in a particular historical context. The financial crisis and its political fallout are parts of this context, while the European refugee reception crisis is another. This latter crisis is both harder to date and define than the GFC. The term is used to refer to an increasing number of people on the move from the Global South to the Global North starting in 2011 and continuing. However, mobility has been a feature of globalized social and economic relations for some time; the features that make this a crisis have to do with the scale of movement and the growing proportion of unauthorized migrants amongst those who are entering the EU. In 2010, the World Bank estimated that about 216 million, or about 3.2% of the world's population was on the move; of these between 10 to 15% were unauthorized migrants (Tilly, 2011). By 2017, this figure had increased to 244 million (United Nations Report on Sustainable Development Goals, 2017). An additional contributing factor was the GFC itself, in that economic austerity meant less investment in and underfunding of the social programs necessary for responding to these increased flows of people (Trauner, 2016).

However, the term “crisis” only came into more common use following significant media coverage of the tragic deaths of migrants attempting to enter the EU via the Mediterranean Sea The term soon came to connote not just the risks inherent in the crossings but also the risk to Europeans and European institutions posed by migrants once they arrived (Holmes and Castañeda, 2016). As many scholars have noted, this rendering of the movement of people across borders and into the European Union has fostered further representations of migrants as dangerous, undeserving, and of posing an unsustainable burden on the social services of receiving nations (Crawley and Skleparis, 2018).

In fact, use of the term crisis for both sets of events is revealing on many counts. For instance, as many authors have pointed out, framing the GFC as a crisis imbues it with externally contingent and unforeseen qualities. This is not born out by the evidence, which, in contrast, suggests that the GFC was both predictable, expected and an emergent outcome of policy decisions made within relevant regulatory regimes (Helleiner, 2011; Keen, 2013). It is possible to make similar observations with regards to the refugee reception crisis; whereas there were external triggers that precipitated mass efforts of people to migrate to the EU over this past decade, the “crisis” element of this migration emerged due to inadequacy of asylum policy and infrastructure in receiving countries, including inadequate attention to how EU policy instruments addressed the disparate impact of migration on southern peripheral member states (Trauner, 2016); how the financial crisis had led to an even more fragile and underfunded asylum infrastructure (Trauner, 2016). As discussed above with reference to the GFC, these structural weaknesses were both known prior to 2015 and remediable within European Union regulatory regimes (Guild et al., 2015).

## Citizenship and the Politics of Memory

“*The past is therefore a permanent dimension of the human consciousness, an inevitable component of the institutions, values and other patterns of human society*” (Hobsbawm, 1972, p. 3).

Citizenship is a complex construct and one that defies easy categorization. It is simultaneously a legal status—one which affords mobility rights and civic, social, and political protections—and a normative construct that defines national belonging, is identity forming, and links citizen rights with citizen duties. The particular interest in this paper is in European Union citizenship, which, as a legal and political construct, is associated with the 1992 Maastricht Treaty, and with further normative and legal elaborations in Article 17 of the Amsterdam Treaty (1997) [although Olsen has argued compellingly that EU citizenship was incipient in earlier European Union treaties (Wiener, 1997; Olsen, 2008)]. While the discussion on EU citizenship has focused on its legal dimensions, and less on its normative aspects (Olsen, 2008) more recently there has been increasing attention to the role of EU citizenship in fostering post-national identities (as well as post-national legal rights) in order to understand the challenges associated with European integration (Olsen, 2008; Keating, 2009); that is, how can EU citizenship rights become levers for fostering European integration.

The complex and fraught status of EU citizenship in the EU today has generated considerable academic and policy attention (see for instance Isin and Saward, 2013). For the purposes of this paper—that is understanding the relationships between discussions of EU citizenship (especially as an integrative identity and status) within the context of austerity policies and the fraught memory politics of today—it is necessary to consider what EU citizenship means, in terms of national identity, national citizenship, and European memorialization projects.

National identities are importantly the products of memorialization projects—they emerge through complex social projects that draw on constructions of the collective experiences of the present as well as collective memories (memorialization) of the past to create national imaginaries (Smith, 1992). Collective memory is simultaneously a body of knowledge about a culture, an attribute of that culture as well as the process by which it is formed (Halbwachs, 1992; Dudai, 2004; Wertsch and Roediger, 2008). Collective memory is about what we know about our past(s), how we know it, and why; and, as with all collective engagements with the past (whether through memory or history), social memory reflects and shapes our understanding of our present moment (Hobsbawm, 1972). The social memorialization of the past is thus inherently a political act (Olick and Robbins, 1998). Beyond this, memorialization of the past, as an ongoing, engaging, and emotionally intense social project, builds national identities, and, importantly, conceptions of citizenship (Habermas, 1996).

To the extent that there is an identity associated with European Union citizenship, it is equally embedded within collective memorializations. These are aspirational and normative memory politics that call forward a concept of a European identity that is both pluralist and pan-European (Smith, 1992; Holmes, 2009). This citizenship identity draws on a mnemonic community that is centered around accepted memorializations of the Holocaust, the Second World War, and the aftermath of these events (Mälksoo, 2009; Rigney, 2012). These memorializations counterpose the notion of a divided past against the integrated present; value universalism over fragmentation; and locate nationalism and xenophobia as part of a shared traumatic European past. Thus, the shared history of World War II and the memorialization of the Holocaust provide both meaning and content to European Union citizenship identities while also challenging ethno-nationalist constructions of citizenship (Rothberg and Yildiz, 2011; Rigney, 2012).

Like national citizenship identities, EU citizenship identities must be concretized through legal and regulatory regimes. EU citizenship, as a set of legal rights, is not bounded by the nation-state, but rather, by that state's membership in the European Union (Bellamy, 2004). For some, EU citizenship expresses a new form of post-national citizenship (Tambini, 2001) that is layered on top of national citizenship. For Holmes, EU citizenship is a form of experimental identity that requires a constant navigation of the meanings and experiences of a pluralist Europe (Holmes, 2009).

However, European Union citizenship and citizenship identities are perhaps less post-national in practice than in theory, however. To begin with, as argued by Hansen, the extent to which EU citizenship is truly post-national may be more about the thinness of the citizenship rights that it is able to confer, rather than the actual post-national rights that EU citizenship confers (Hansen, 2009). In other words, EU citizenship has not dislodged the role of national citizenship as a key determinant of legal rights. As a legal status, EU citizenship complements national citizenship; thus existing differences in citizenship rights between EU member states remain even as EU citizenship is articulated as a series of fundamental human rights.

EU citizenship identities—as non-particularistic and as distanced from the ethno- national state—are also contingent and fraught, as seen through the recent emergence of new nationalist politics in the EU. These new politics are based in memory politics around new (or re-discovered/re-articulated) mnemonic European communities; these re-articulate and re-define the relationship of shared history to belonging, by, for instance, placing language, identity, and place at the forefront of citizenship identities (Misztal, 2010). These new movements importantly draw on an understanding of the failure of post-nationalism, as offered through European Union integration (Zaslove, 2008). In other words, the politics of memory continue to be central to the construction of citizenship and to the exercise of citizenship rights in the EU today.

Clearly, the relationship between citizenship and the politics of memory is well worth interrogating. Citizenship is the anchoring concept of the nation-state. The development of citizenship as a global, universal status, and one that puts, at least in principle, principles of equality at its forefront, is the foundation of contemporary democracies. As argued by Marshall (1950) robust and “thick” citizenship—that includes not just civic freedoms, but also social rights and economic protections—provides the basis of political equality as well as for social cohesion and sustainability, and political and social stability (Marshall, 1950).

While Marshall cited universalism as one of the fundamental attributes of citizenship, in practice, citizenship is both particularistic and differentiated (Soysal, 2000) and generative of deep inequalities (Shachar, 2009, p. 130–141). Citizenship, in particular, involves drawing lines: Birthplace, gender, race and ethnicity, language, and legal status all interplay to create hierarchies and inequalities between and amongst citizens (O'Connor, 1993). For these reasons, it is perhaps not surprising that there has been generally more scholarly attention to citizenship's potential to create inequalities than the reverse (Shachar, 2009). For instance, inequalities around birthplace, race and ethnicity have been shown to be associated with significant “thinning” of citizenship rights for immigrants and migrants (Brysk and Shafir, 2004; Schierup et al., 2006; Dell'Olio, 2017). In addition, legal regimes around work and mobility create significant differentiations, especially between migrants and residents, and produce both precarious and contingent citizens.

At the same time, the theoretical and historical legacy of the concept of citizenship also has a strand of inclusionary and integrationist concerns. For instance, as per Marshall's more hopeful articulation, citizenship was the foundation of equity in the post-World War economic order (Marshall, 1950). This dual legacy suggests that citizenship can carry the seeds of integrative discourses just as it can carry exclusionary discourses. Hannah Arendt's thinking around the communal roots of citizenship is particularly helpful here. Arendt begins with the premise that co-existence with others like us and not like us—living collectively and in communities—is fundamental condition of humanity. Further to this, all meaningful human rights to existence (i.e., life) are guaranteed by membership in a community. Thus, for Arendt, the genocide of the European Jewish community during World War II was possible because Jews had been deprived and removed from community—from “a place in the world” (Arendt, 1985, p. 268). The *right to have rights* is the fundamental premise of citizenship; the right to have rights requires a community that gives content and meaning to those rights (Arendt, 1985).

This emphasis on the communal dimensions of citizenship reinforces also the relationality of citizenship. Citizenship is embedded in and re-embeds social relations of mutual obligation: paying taxes, military service, voting, obeying the law (on the part of the citizenry) in exchange for the provision of services by the state, protection from enemies, and freedom from illegitimate exercise of violence (Somers, 1993; Joppke, 2007). The relations of citizenship extend into social, civil, and cultural realms, to include not only economic participation (i.e., the worker-citizen), but also social reproduction (the mother-citizen) (Stoltzfus, 2004; Hallgrimsdottir et al., 2013; Anderson, 2015).

Citizenship is thus a complex and multi-layered concept that involves not only the rights that flow from the legally recognized residence within a particular territory, but also identities and statuses that accompany those rights as well as the processes and actions that realize them (Marshall, 1950; Turner, 1990; Somers, 1993; Soysal, 2000; Isin and Turner, 2007; Isin and Nielsen, 2013). Citizenship's complexity is seen at one level in the fact that it can be wielded as both a weapon of exclusion as well as an integrative tool. However, at its foundation, citizenship is a normative construct that expresses equality as a social good (Somers, 1993, as per Arendt, citizenship, at its best, expresses and rearticulates communities, sustains the autonomy of civil society and safeguards against the tyranny of the state; Arendt, 1961). Citizenship conceived of as community-derived rights that draw on responsibility, mutualism, and political engagement has thus the potential to recast the conversation away from the *differentiation* of rights toward *universalism*.

However, it is clear that in our current moment, universalism is not at the forefront of citizenship narratives in the EU. Rather, citizenship is thinning and attenuated (Stasiulis, 2004), and fraught with nationalist concerns. As elaborated further below, the language of crises—cultural, political, and economic—presents a significant normative challenge to universalism in citizenship narratives. Instead, narratives of crises, given new content by alternative memorialization of Europe that draw attention to identity, culture, language, and place, give rise to attenuating and exclusionary narratives of citizenship; narratives that, as argued by Nyers, foster “forms of governance that are responsive to—and constitutive of—fears, anxieties, and insecurities” (Nyers, 2009).

## Narrating the European Union Through Crises

The notion of crisis as a trigger of great change has a long historical legacy. In fact, the very idea of the Union is itself a product of crisis: the European project was triggered by the economic and political disarray that followed the Second World War (Dinan, 2004). More generally, crises play a key role in epistemologies of social change. Crises are *contingent* events—events that alter path-dependent patterns of historical change (Pierson, 2000). Crises are also understood to generate critical junctures that force new choices or decisions upon institutional actors (Peters et al., 2005; Capoccia and Kelemen, 2007). Scholars of public policy often conceptualize crises as the “external shocks” that can shift policy solutions from incremental to substantive (Jenkins-Smith and Sabatier, 1999; Nohrstedt and Weible, 2010).

Crisis operates simultaneously on multiple registers: as a theoretical or methodological lens, as an empirical descriptor, and as a normative and narrative device. In the European Union context, crisis has a long empirical and theoretical history. The European integration literature is dominated by functionalist and neo-functionalist perspectives that place crisis at the core of European integration: the European project itself was triggered by the economic and political disarray that followed the Second World War (Dinan, 2004; Dines et al., 2015). In addition, initial steps toward integration triggered further, smaller economic and political crises, that were then solved through further integration, through for instance policy parallelism or economic and political unification (Lefkofridi and Schmitter, 2014). The notion that crises are necessary for creating systemic change is rooted as well in Marxist and post-Marxist theories of large-scale social and historical change. James O'Connor, for instance, expands on and develops the role that economic and ecological crises play in terms of restructuring the social relations of production in a post-capitalist context (O'Connor, 1988); crises foster contradictions that need to be resolved in order for the system to sustain itself.

Crises need however to be identified as such in order to effect historical change. The meaning and directions of crisis is driven by context, and of course, the decisions and actions of human actors. Gramsci reminds us that on their own, economic crises cannot cause political change, but that rather they “simply create a terrain more favorable to the dissemination of certain modes of thought, and certain ways of posing and resolving questions involving the entire subsequent development of national life” (Gramsci, 1992, p. 276). Crises are always simultaneously narrative devices and constructs as much as they are historical “things.” As discussed above, the European migrant crisis, for instance, was as much a crisis of European migration policy, of European citizenship, and of European identities, as it was a crisis of migration: the valences and meanings of the migrant crisis were clearly driven by national and domestic political agendas (Berry et al., 2016).

As a narrative device, the concept of crisis is often nested in dystopian, even apocalyptic understandings of events: the future is both uncertain and unknown. Gramsci's definition of crisis as “The old is dying, and the new cannot be born,” (Gramsci et al., 1971, p. 276) captures how crisis can take on opposing valences, as simultaneously a moment of openness and possibility and risk, danger and uncertainty. Crisis as a narrative calls forth action and immediacy; in the EU as well as in North America, positing the flow of migrants as a crisis has justified moments of exception to the rule of law, and the suspension of human rights (Hansen and Stepputat, 2005; Dines et al., 2015). The use of camps, detention zones, and expended border zones within which migrants are reduced to “bare life” (Agamben, 1998) within and inside established and wealthy welfare states, such as Italy and France, illustrates in part the power of a crisis narrative to suspend normal modes of state operation.

There is an additional difference between the responses to this most recent economic crisis and the kinds of responses that earlier crises in the European Union engendered, and that is the shift away from Keynesian economic responses and toward austerity economics as an almost hegemonic response to economic downturn (Lefkofridi and Schmitter, 2014; König and Wenzelburger, 2017). In fact, to the extent that there have been coordinated policy responses to the crisis, these have largely been driven by an austerity agenda (Karanikolos et al., 2013). This is important because austerity politics have in general led to poorer social and health outcomes, but also generated declining trust in politics and in public institutions (Lefkofridi and Schmitter, 2014). In addition, while the financial crisis was instigated by increased economic inequality across Europe, as well as increased economic polarization (Galbraith, 2011; Stockhammer, 2015; Cynamon and Fazzari, 2016) there is an emerging consensus that the policy responses to the 2008 economic crisis have, in general, exacerbated these trends (Vaughan-Whitehead, 2011). Of particular importance from a European perspective is the variegation of these effects, seen in terms of increasing within and between nation economic inequality (Vaughan-Whitehead, 2011). Why states continue to embrace austerity measures in spite of little evidence of their ability to engender economic growth is certainly confounding (Blyth, 2013; König and Wenzelburger, 2017) as are recent (and counterintuitive) findings that suggest that the political risks to imposing austerity measures are lower than expected (König and Wenzelburger, 2017; Arias and Stasavage, 2019).

In sum, the link between crises and integration in our current context is far from clear or certain. As Polanyi has argued, political, economic, and social change only comes from conscious human decisions (Polanyi and MacIver, 1944). In turn, a crisis instigates change through necessitating new kinds of decisions and paradigmatic shifts (Block, 2003). The logic of economic crisis in neoliberal regimes suggest that there is no endogenous mechanism by which crisis will trigger integrative reactions. Rather, if crisis is to trigger further or deeper integration, it will need to be explicitly made by policy actors. But the larger point here, is that whether seen as disruptions to stable systems or as necessary triggers of progressive change, or the catalyst to societal transformation, in these readings crises *make history*. This understanding of crises is important to thinking through how crises as a narrative device function in political rhetoric.

## Crisis and the (Re-)making of History? Narrative Framings of the GFC and the Refugee Reception Crisis

The paper now turns now to a brief exploration of the narrative framings of the GFC and the refugee reception crisis by three key right-wing, Eurosceptic parties. The goal with this analysis was to examine how these two crises have been taken up in Euroskeptic politics, in other words, how have framings of these two crises contributed to and formed new memorializations of both the past as well as the current European moment, and second, how have these crises informed the narratives of European citizenship presented by these parties. The authors examined publicly available policy manifestos of the United Kingdom Independence Party (UKIP, 2018), the Rassemblement National (National Assembly—or what was previously known as the *Front National)* (Rassemblement National, 2017)^2^, and the Alternativ fur Deutschland (AfD, 2017) (Alternative for Germany) that were available on-line in July and August 2019 and that were developed for dissemination for both national elections (in France and Germany) and the European Parliament Elections of 2019. These parties were chosen to represent mainstream Euroskeptic politics in the three largest EU member nations at the time of the analysis (the United Kingdom is scheduled to leave the European Union on January 31st 2020).

Party manifestos were downloaded in their entirety. The documents were translated and analyzed by one of the co-authors of this article. The analysis was conducted as a thematic narrative analysis (Butler-Kisber, 2010): the documents were read initially to develop broad themes, and then iterative analysis was used to narrow down the themes and recategorize. The central themes uncovered (as detailed further below) revolve primarily around scarcity of resources, the dangers associated with immigration, and a consequent need to reframe citizenship. However, it is important to note that this analysis is intended as an empirical elaboration of the discussion above as opposed to a fulsome exploration of memory politics in the political narratives of the European far-right, which would require a larger cross-section of political platforms, including platforms of left-wing Eurosceptic parties.

The analysis reveals two interrelated themes: a sense of crisis in the present and a particular kind of historical consciousness that frames notions of citizenship. In broad strokes, the present is seen as marked by a scarcity of resources blamed principally on immigration. The solution to scarcity for the far right in these cases lies in the restriction of access to public services to those belonging to the “true” national community. This sense of crisis works in tandem, therefore, with a politics of memory that underpins notions of European citizenship in such a way as to identify particular individuals and groups (primarily Muslim immigrants) as incompatible with European culture and providing, therefore, the justification for the subsequent removal of their “right to have rights.”

The key narrative of crisis evident in these policy documents is that of a scarcity of resources apparently caused by dangerously high levels of immigration. In particular, these parties base the survival of valued public services on the exclusion of certain groups from access to these services. For example, UKIP sees the NHS as in crisis in large part due to “ever-increasing demand from foreign nationals who should have no entitlement to use its services free of charge. (UKIP, 2018, p. 2). The AfD, similarly, claim that “*we are merely experiencing the beginning of a gigantic mass migration toward European countries….[t]he European right to freedom of movement has led to massive migration from poorer EU countries to the richer ones, especially to Germany, for the sole purpose of obtaining social aid*” (AfD, 2017, p. 58, 60). Furthermore, they argue that these immigrants frequently turn to crime when their hopes for success in Germany flounder (AfD, 2017, p. 63). Moreover, “austerity measures… have resulted in a massive drain in personnel, which have led to irresponsible and untenable deficits” (AfD, 2017, p. 24). These two trends (austerity and immigration levels) act in tandem to create a crisis in national security and social services in Germany. UKIP echoes these kinds of concerns around the link between Muslim immigration and crime: “[t]he worst excesses of a literalist interpretation of Islamic doctrine has seen unprecedented acts of terrorism in Britain and across the world” (UKIP, 2018,p. 14). In France, the RN represents the current crisis in similar ways—as the breakdown of public order. This is attested to by the numerous proposals in their policy manifesto calling for a “massive” increase in the “forces of order,” including an increase of 15 000 police officers and gendarmes (Rassemblement National, 2017, p. 5). That immigrants (and citizens seen as not “truly” French) are to blame for a large proportion of violence is evidenced both in policies calling for the augmentation of border controls, the restriction of immigration to 10 000 per year, the restriction of the acquisition of citizenship to birth and naturalization by more stringent conditions (Rassemblement National, 2017, p. 6). The central task for these parties, therefore, is to define their national community in such a way as to exclude certain groups.

Key to these discourses is a specific historical view of what constitutes a “true” citizen of a given nation. For these parties, the only important condition for citizenship is cultural/historical. The AfD captures these conditions neatly and explicitly: “[German] culture is derived from three sources: firstly, the religious traditions of Christianity; secondly, the scientific and humanistic heritage, whose ancient roots were renewed during the period of Renaissance and the Age of Enlightenment; and thirdly, Roman law, upon which our constitutional state is founded” (AfD, 2017, p. 46). In the RN's policy manifesto, the history of France and the French language are named as two of three fundamental topics of study (along with mathematics), and schools are asked to dedicate 50% of class time to the teaching of the French language and to eliminate the teaching of “languages and cultures of origin” (Rassemblement National, 2017, p. 16). A shared language and history, therefore, are the two key features of French citizenship for the RN, a national culture and identity that, according to them, must be defended and promoted in the constitution itself (Rassemblement National, 2017, p. 15). UKIP rhetoric echoes these themes as well. While English identity “resides in the heart and mind not on the skin,” it is nonetheless an exclusionary identity in UKIP's formulation. UKIP advocates for the implementation of a system where only MP's representing English constituencies are allowed to vote on laws affecting England (UKIP, 2018, p. 16). Furthermore, UKIP sees the English identity as under attack, as being “airbrushed out of our national life” by an “anti-English minority …over represented in the institutions of government, politics, the leadership of the public sector, the media, corporate capitalism and academia” (UKIP, 2018, p. 15).

Crucially, the community of citizens constructed by these narratives is hostile to other cultures: “[t]he ideology of multiculturalism is blind to history and puts on a par imported cultural trends with the indigenous culture, thereby degrading the value system of the latter” (AfD, 2017, p. 47). German culture is, therefore, “degraded” by the mere presence of other cultures in proximity. Similarly, the RN frames its defense of the rights of women in terms of opposition to “Islamism” (Rassemblement National, 2017, p. 4). French values around human rights are framed as under attack by other cultures. The UKIP groups policies in its manifesto dealing with the condemnation of “Sexual Exploitation & Pedophile Gangs” (blamed primarily on Muslims) and “Islamic Extremism” in the section entitled “British Culture” (UKIP, 2019). This association makes obvious the link drawn by UKIP between Islam/Muslims and violence. Islam is framed in all three cases as inherently incompatible with Western values—creating a separation within Western nations between individuals who are seen to be “true” citizens and those who are not (those from other cultures). A deputy for the RN captured this sentiment exactly when commenting on celebrations in French cities following Algerian victories in the 2019 African Cup of Nations: “there exists a people within a people. People that, under the cover of sport, defy the state that they do not consider their own.” (Rassemblement National, 2019) is the key anxiety underlying all three parties' platforms; that there exists, within the national community, a fifth column (in all cases the primary target are Muslims) who hold views seen as incompatible with Western values, who are draining the prosperity of the nation through demands on public services (most importantly public order), and who must, therefore, be excluded from citizenship. The central rhetorical move of the far-right, as seen in these platforms, is that of differentiation. Cultures are represented as inherently incompatible, and to attempt to integrate them is to provoke crisis. The language of crisis, then, plays a crucial role in providing the justification for citizenship based on differentiation; crisis acts as the catalyst for change, provoking the necessity for a narrativization of history that produces exclusionary narratives of citizenship.

The narratives of these three far-right parties share a common thread: crises that provide the justification for a further restriction of citizenship rights to those who belong to the right kind of community based on a particular historical and cultural heritage. Importantly, this specific community is defined based on a particular reading of history within European Union member states—one which emphasizes the uniqueness of Western values and their inherent incompatibility with outside cultures. In this way, politics of memory are deployed by far-right parties in intersection with narratives of crisis to produce a “thinning” of citizenship—a move from universalism to differentiation based on the politics of memory. The language of crisis acts as a narrative device for producing binary oppositions: West vs. East, civilization vs. barbarity, Western values vs. Islamic extremism, true German/French/English citizens vs. immigrants, and so on. The key task facing the European Union, and in particular European integration, therefore, is to reorient the language of crisis to provoke positive change, to definitively embrace the principle of universalism and to resist definitions of citizenship (like those discussed above) that take as their starting point the existence of essential and irreconcilable differences between cultures based on their history.

## Discussion and Conclusions

Crises are the key narrative device of our time. As a narrative device, it is a double-edged sword: while framing historical events as crises denotes urgency and the need for action, this also creates totalizing narratives that fosters polarized binaries. Narratives of the migrant crisis rested on binary understandings of citizens vs. non-citizens; deserving vs. non-deserving migrants; and importantly, rule of law vs. lawlessness. In turn, the migrant crisis narrative was clearly framed by the narratives emerging out of the GFC: the need for austerity, the scarcity of resources needed to support the social good; the unsustainability of rich welfare supports. In this way, narratives of migration are hooked into narratives of citizenship. In our current context, these citizenship narratives are fueled by a rich resource of “forgetful” renderings of the past. These narratives were in fact accompanied by real and significant action of the state, resulting in noticeable withdrawal of the state from providing social services and a general disassembling of the welfare state.

The power of the economic austerity narratives that took hold after the GFC can, at some level, be seen by examining social and political changes in European Union nations that were the least affected by the financial crisis, such as Sweden and Norway (Finnsdottir and Hallgrimsdottir, 2019). Both of these nations have seen a resurgence in support for right-wing populist parties with anti-immigration agendas, in spite of not having suffered the kind of economic set-back that has fuelled the rise of left- and right-wing populism in Greece, France, Spain, and Italy.

In fact, the narratives of economic restructuring and recovery that emerged out of the GFC were equally polarizing and totalizing as narratives of the migrant crisis. While the GFC was a complex historical event with an equally complex etiology, as a narrative, the GFC is a relatively simple story of victims and villains, that links together national debt with profligacy and moral failure. In this story, austerity and cut-backs to social spending are valorized as strength and determination; the political agency of state actors is in effect minimized, as there is in effect only one plot-line, one solution. As a general comment, the very casting of the GFC as an economic story, as opposed to a story of political action, inaction, and failures, is interesting in and of itself.

In terms of the discussion above, as evidenced by how these crises were deployed in the political narratives of Euroskeptic parties, the GFC can be linked to a *thinning* of citizenship while the migrant crisis can be linked to a *thickening* of borders. In this, the political platforms of these parties illustrate that both a more exclusive as well as more contingent basis for social inclusion, cohesion, and integration in the European Union. Taking into account the electoral success that was enjoyed by each of these political parties in recent elections (the AfD garnered 11% of the vote in the 2019 EU parliamentary elections nationally, but with a stronger showing in east Germany; the RN received 23% of the popular vote in France; the Brexit party (a successor to UKIP, received 30% of the popular vote in the United Kingdom) these narratives cannot be seen as incidental to debates about European integration as well as citizenship in the EU (European Parliament, 2019). Putting aside the extent to which universalism was actually realized in the legal and political exercise of European Union citizenship, these framings show how universalism as *normative* project of European integration has clearly been significantly challenged over the past decade.

However, seeing crises as sets of contingencies and catalysts that trigger historical change, is also suggestive that both the GFC and the migrant crisis could have been cast as opportunities and openings to new ways of social and economic organization. Certainly, the Occupy movement of 2011 attempted to draw on the disruptions of the GFC in order to instigate new conversations about economic inequality. While the reasons for the failure of Occupy to create meaningful change are beyond the scope of this paper, it is worth noting that one of the challenges faced by the Occupy movement was the lack of institutional infrastructures that were key to the successes of earlier civil and social uprisings (from the McCarthy and Wolfson, 1992; Calhoun-Brown, 2000). In particular, political parties, unions, and civic associations that provide networks, formal and informal relationships, and cohesion to claims, and give meaningful collective identity, shared goals, and membership in mnemonic communities, to protestors and social activists. Drawing on Arendt again, it is possible to hypothesize that the Occupy protesters protested without clear reference to a membership in community. Occupy was protesting neo-liberalism, but did so within a context of the already thinned fabric of citizenship and community created by neo-liberalism.

Crisis narratives are catalyzing constructs. This is evidenced by the embrace of the word crisis to describe ecological and climate emergencies. In this context, activists use crisis narratives so as to justify *new* solutions to pressing problems and to discount the efficacy of old solutions. Yet, both the GFC and the refugee reception crisis were largely met by new iterations of old policy solutions. Clearly, the extent to which crisis acts as a contingent event is thus dependent on a range of external factors. In the instances examined here these crises fostered and gave credence to thinned and truncated narratives of citizenship within the platforms of mainstream Euroskeptic politics.

## Data Availability Statement

The datasets generated for this study are available on request to the corresponding author.

## Author Contributions

HH wrote the initial full draft. AF was responsible for collecting the empirical data. EB-J edited, revised, and contributed to theoretical framing of the article.

### Conflict of Interest

The authors declare that the research was conducted in the absence of any commercial or financial relationships that could be construed as a potential conflict of interest.

## Footnotes

^1^The term refugee reception crisis is used to indicate that the crisis was caused by lack of capacity and incapacity of receiving countries, rather than by the migrants themselves.

^2^The Front National was renamed the Rassemblement National on June 1st 2018.
